# Radiation-based quantitative bioimaging at the national institute of standards and technology

**DOI:** 10.4103/0971-6203.54843

**Published:** 2009

**Authors:** Lisa R. Karam

**Affiliations:** Ionizing Radiation Division, National Institute of Standards and Technology, 100 Bureau Dr. MS 8460, Gaithersburg, MD 20899-8460, USA

**Keywords:** Computed tomography, dosimetry, imaging, phantoms, positron emission tomography, treatment planning

## Abstract

Building on a long history of providing physical measurements and standards for medical X rays and nuclear medicine radionuclides, the laboratory has expanded its focus to better support the extensive use of medical physics in the United States today, providing confidence in key results needed for drug and device development and marketing, therapy planning and efficacy and disease screening. In particular, to support more quantitative medical imaging, this laboratory has implemented a program to provide key measurement infrastructure to support radiation-based imaging through developing standard, benchmark phantoms, which contain radioactive sources calibrated to national measurement standards, to allow more quantitative imaging through traceable instrument calibration for clinical trials or patient management. Working closely with colleagues at the National Institutes of Health, Rensselaer Polytechnic Institute, the Food and Drug Administration and Cornell University, this laboratory has taken the initial steps in developing phantoms, and the protocols to use them, for more accurate calibration of positron emission tomography (PET) or single-photon emission computed tomography (SPECT) cameras, including recently standardizing ^68^Ge. X-ray measurements of the laboratory's recently developed small, resilient and inexpensive length standard phantom have shown the potential usefulness of such a “pocket” phantom for patient-based calibration of computed tomography (alone or with PET) systems. The ability to calibrate diagnostic imaging tools in a way that is traceable to national standards will lead to a more quantitative approach; both physician and patient benefit from increased accuracy in treatment planning, as well as increased safety for the patient.

## Introduction

This laboratory provides the physical measurements and standards in a variety of fields, including for ionizing radiation for healthcare applications for the United States. Since the mid-1920s, our standards have been addressing the need for the measurement of exposure from conventional diagnostic X-ray beams used in some 300 million procedures per year in the United States.[1] Radioactive standard reference materials (SRMs) developed and produced, and source calibrations performed, in this laboratory provide traceability for radiopharmaceuticals, including many used in medical imaging. Focus has been expanding to address measurement standards to support the quantitative potential of imaging technology as a potential to identify early signs of disease (biomarkers), provide early indications of drug response and measure therapeutic changes.

The measurement standards foundation of ionizing radiation in the United States is a mission carried out by three research groups within the larger Division. One group develops dosimetric standards for X rays, gamma rays and electrons for homeland security, medical, radiation processing and radiation protection applications, which are based on the *Système International* (SI)-derived unit, the *gray*. Another group develops and provides neutron standards and measurements needed for fundamental physics, homeland security, the hydrogen economy, worker protection and nuclear power. The third group develops and provides standards for radioactivity based on the SI-derived unit, the *becquerel*, for homeland security, environmental, medical, and radiation protection applications. The Division pursues several avenues to provide the measurement infrastructure needed by the community. Calibrations (instruments calibrated against standards can be compared with one another) of artefacts (e.g., instruments and radiation sources) and transfer standards allow the community to submit specific requests to this laboratory. Radiation and radioactivity standards (SRMs and quality/calibration factors) distributed by the Division to the community enable consistent measurements across research and clinical centers. Measurement assurance (through traceability programs and cooperative research) provides the opportunity for the laboratory and stakeholders to work together to address measurement needs affecting the wider community.

Although the Division's efforts impact a variety of industries (automotive, defence, environment, fundamental science and research, homeland security and industrial applications), recent efforts have expanded a focus on healthcare, particularly on medical imaging. This expansion is supported by expertise in radiation physics (theoretical dosimetry, codes and modelling), dosimetric measurements (X-ray calibrations, brachytherapy and mammography) and radioactivity measurements (radionuclide standardization, standard reference materials and calibrations). Previous medical imaging work demonstrates how measurement standards can support regulatory needs. In 1992, the US Congress enacted the Mammography Quality Standards Act (MQSA) to assure that women would have access to quality mammography for the detection of breast cancer “in its earliest, most treatable stages.”[2] Enforcement of the MQSA required that mammography instrumentation in the United States met strict standards of radiation exposure and energy characteristics as regulated by the Food and Drug Administration (FDA). To achieve this goal, the laboratory and the FDA developed the necessary calibration facilities at this laboratory, including the US national standard for radiation exposure from X-ray beams used in mammography, which is used for calibration of exposure meters in these beams in proficiency testing of instrumentation used throughout the country.[3,4] Four instrument manufacturers and the FDA itself, as well as three dosimetry calibration laboratories, accredited by the American Association of Physicists in Medicine, now meet the legal requirements of the MQSA. The accuracy of radiation exposure measurements in more than 13 000 US mammography facilities can now trace back to the US national measurement standard. Accurate assessment of radiation exposure is crucial not only for patient safety but also to assure image quality and minimize the need for repeated exposures.

Medical imaging gives physicians and researchers an insight into the body, even cells themselves, and has been one of the most important medical developments of the past 1 000 years. It provides a crucial tool for early, noninvasive cancer detection, monitoring of treatment efficacy, drug development and lower-cost healthcare. Historically, medical imaging has been mostly qualitative, good for “yes/no” answers. However, semi-quantitative results give semi-reliable answers and many healthcare applications, such as treatment planning, patient evaluation, drug development and clinical studies, demand a more quantitative approach for assessing efficacy and for patient safety. Patient concerns (“Do you see it? What is it? Where is it? Has it changed? What's it up to? Just how much radiation do I have to get?”) can also be best addressed by a more quantitative approach to medical imaging. Recognizing these issues, representatives of the drug and medical imaging industries, FDA, National Cancer Institute (NCI) and various professional organizations such as the Radiological Society of North America, approached this laboratory to provide physical standards and calibration tools for quantitative imaging (computed tomography [CT], positron emission tomography [PET], magnetic resonance imaging, spiral CT, bone health, optical imaging) and measuring therapy-induced change as well as standards for image generation, transmission, archival storage and dissemination to researchers.[5]

## Methods and Results

To support more quantitative medical imaging, standard (or traceable to national measurement standards) phantoms are needed to calibrate a variety of instruments, particularly for radiation-based methods such as PET, CT and single-photon emission CT (SPECT). In PET or SPECT, particularly because the radionuclides used in these modalities can be very short lived, it has been nearly impossible to provide such traceable measurements for the phantoms that are currently used to calibrate these types of instruments. Initially, then, efforts were focused on developing tools to enable quantitative imaging with these radiation-based technologies.

The most significant advance in CT technology in the past few years has been the development of spiral (or helical) CT, an emerging method to detect small changes in the volume of a tumor more rapidly and often with less radiation dose than conventional CT. Spiral CT is used to evaluate and diagnose pulmonary embolism, cancer, treatment efficacy (tumor size changes), diseases of the circulatory and digestive systems. Changes in the order of 30% (for pea-sized tumors) to 10% (for grape-sized tumors) are of particular interest in the study of tumor drug response to assess therapy effectiveness and for drug development. In fact, use of spiral CT in the United States has increased (and continues to increase) dramatically in recent years (to approximately 60 million procedures/year). The reliability of these volume assessments depends on both physical and biological knowledge and measurements. Although the evaluation of X-ray interaction physics with tumor mass models is crucial in determining the subtle effects such as X-ray scattering might have on measurements, many discussions with the user community indicated that a higher need was for a low-cost length standard phantom representing tumors *in situ*. Rather than attempting to mimic the physical manifestation of tumor development, such a phantom would be useful for instrument performance evaluation and to calibrate commercial CT scanners for each patient, compensating for instrumental drift, so that different instruments may be used over time. Such a phantom could be used in the evaluation of algorithms for volumetric measurements, eventually leading to improved patient care (by reducing the need for repeated procedures with associated increases in dose and patient inconvenience as well as additional healthcare costs) and improved determination of treatment effectiveness.

**Figure 1:**
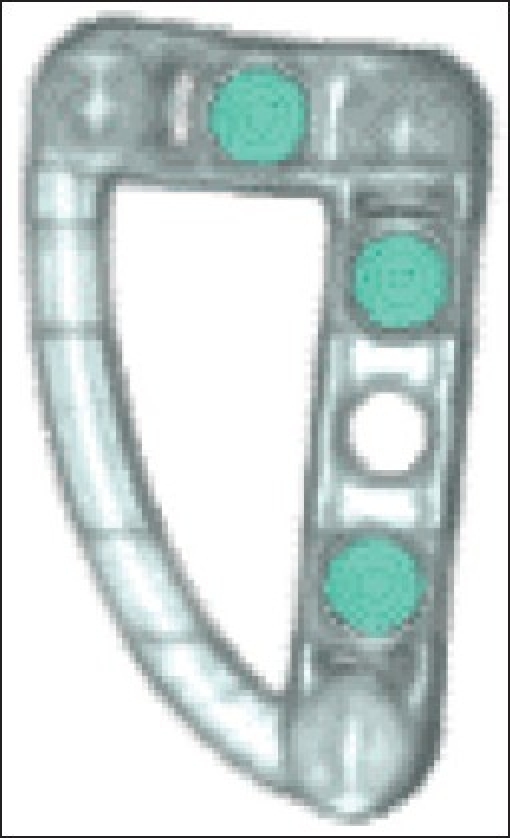
3D visualization reconstructed from CT images of fiducial reference “pocket-phantom” designed and constructed at this laboratory (from Anthony P. Reeves, Cornell University, Ithaca, NY; personal communication)

Cooperation and collaborations with a variety of stakeholders (including Cornell University, the FDA, the national Institute of Biomedical Imaging and Bioengineering (NIH-NIBIB), the Society of Nuclear Medicine (SNM) and others) to facilitate development of realistic benchmark standards for validation provided crucial input as to the requirements of a potential “length standard” phantom for medical CT. During several meetings with stakeholders, particularly with David Yankelevitz and Tony Reeves from Cornell University, specific requirements of such a phantom were expressed (that it be very inexpensive, portable and/or patient-specific, and traceable to national standards to facilitate clinical trials and FDA applicability). Working with these colleagues from Cornell, as well as with others from industry, we designed a plastic-based fiducial reference CT phantom, or “pocket-phantom,”[Figure 1] which is inexpensive, robust and provides good contrast.[6] The plastic fixture (about 3.5 cm in length) absorbs X rays similar to water, and is near tissue equivalent, with an attenuation of approximately 0 Hounsfield units (HU). The small spheres contained within the fixture (turquoise color in the figure), when made of Teflon*, have an attenuation of approximately +800 HU (±25%). Glass spheres also show good contrast with the fixture, while other materials tested (Delrin, Acetyl, Torlon) do not. This design has received positive feedback from stakeholders, and plans are underway to implement its use in an upcoming clinical trial.

In addition to CT-phantom development efforts and radioactivity standardizations, phantoms for calibration of the PET component of PET-CT instrumentation are under development. PET-CT is increasingly used to diagnose disease, (especially cancer) and to plan and monitor response to treatment; 1.3 million PET procedures were performed in the United States in 2005, a number expected to increase at a rate of up to 10 % per year (predominately in the dual modality configuration of PET-CT). The usefulness of PET in monitoring depends on consistent patient data over weeks or months, and from one center to another. Attendees at an NCI-sponsored workshop during recent SNM meetings recognized calibrated phantoms and methods for PET instrumentation to be important for improving image consistency and quantification accuracy. A potentially powerful diagnostic imaging tool, PET (alone or with CT) instrumentation traceably calibrated to national standards will lead to more quantitative results for patient assessment, drug development and treatment planning. Developing useable phantoms to calibrate and evaluate the performance of PET instrumentation presents some additional difficulties. Stakeholders from FDA to industry (including drug developers) have expressed the need for traceability in activity measurements for instrument calibration using longer-lived isotopes than those used in conventional PET. In addition, phantoms that more closely mimic the human body, and protocols for their use and training, have been proposed as crucial components to the implementation of quantitative PET-CT.

**Figure 2:**
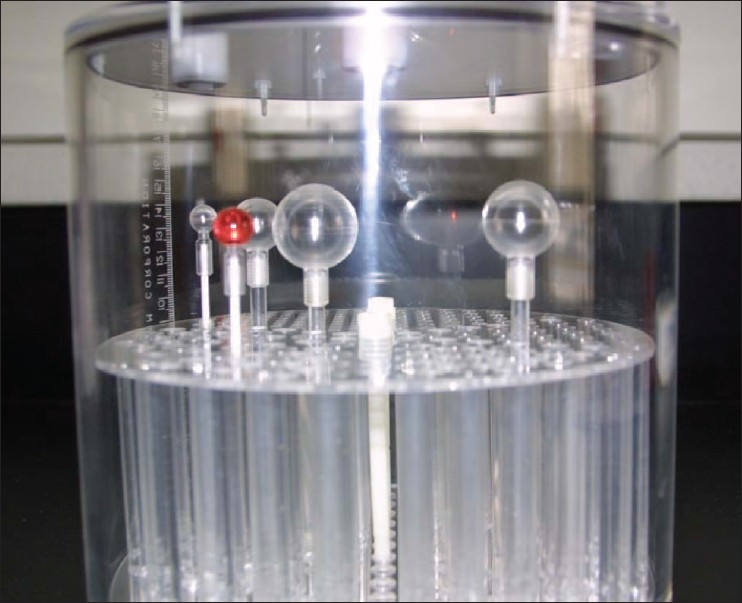
New phantom, designed and constructed at this laboratory, for PET instrumentation calibrations (“cold” epoxy version; will contain Ge-68)

Although the radionuclides used in PET procedures are extremely short lived (one of the most common, ^18^F, has a half-life of about 110 minutes), a longer-lived surrogate must be used in an SRM phantom for PET in order to allow for distribution to most users. Conventionally, ^68^Ge (t_1/2_ = 271 d) has been used to calibrate instrumentation and to assess PET attenuation, but has not been useable as a cross-center reference as measurement of its activity has not been traceable to a national standard, which, until recently, did not exist. In addition, calibration of ^68^Ge in various relevant geometries (eg, syringe, phantom) had yet to be done. In 2008, the first national calibration of ^68^Ge (in equilibrium with its daughter, ^68^Ga) was developed in this laboratory,[7] and we have been working with RadQual, LLC, for developing calibrations in a variety of geometries. The first such geometry, a phantom that could be used for instrumentation calibration[Figure 2], has been developed and is currently undergoing testing. Produced via the incorporation of calibrated ^68^Ge into an epoxy matrix, this phantom would be a relatively stable (over 1 year) artefact that could be used for consistent calibration of and comparisons among PET instrumentation. A calibration for the syringe geometry (in liquid and epoxy) is also underway. The ^68^Ge calibration has been linked back to the national ^18^F standard (which is calibrated for the NIH PET Center and for PETNET Solutions, a regional distributor) in order to provide traceable measurements for injected activity. Compared to measurements of the same geometry of ^18^F in four clinical-style dose calibrators, the response factor (ie, the relative response of a dose calibrator while the setting for ^18^F is used in the measurement of the same geometry of ^68^Ge) for the ^68^Ge in a syringe was determined to be 1.054 ± 0.020; Monte Carlo calculations predict this factor to be 1.053. The primary calibration has been transferred to the laboratory's 4πγ Secondary Standard Ionization Chamber to facilitate more routine future calibrations; because Monte Carlo modeling predicts about a 0.1% difference in ionization chamber response between liquid- and epoxy-filled syringes for ^68^Ge, no correction due to the change in matrix need be applied.

**Figure 3:**
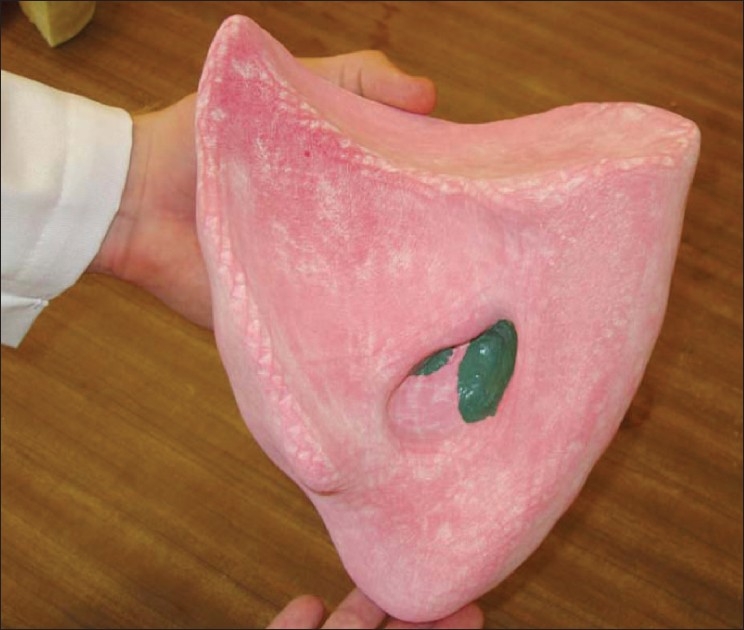
Anthropomorphic, CT image-based lung phantom (from George Xu, RPI; personal communication)

The laboratory has also been collaborating with Rensselaer Polytechnic Institute (RPI) to develop standardized, calibrated anthropomorphic phantoms, beginning with the lung, which was developed from converted CT and MRI data via conversion to 3D mesh models [Figure 3]. When embedded with a standardized ^68^Ge source, such phantoms can be used to assess comparability of data from multiple sites during clinical trials and comparability of reconstruction techniques. Eventually, the groundwork for quality assurance/quality control measurements to assure phantom reproducibility will be developed.

## Discussion and Conclusions

Several follow-up efforts will enable implementation of these standards to meet the needs of the community. The performance (micro CT, gamma ray detector measurements and SPECT or PET image data with computational simulations) of the newly developed CT and PET-CT phantoms will be validated. Geometry, activity level and counting efficiency relationships will be established, and Monte Carlo computations will be validated against measurements. Additional benchmark anthropomorphic phantoms (different sizes/genders) will be fabricated and protocols for accurate calibration of dual modalities (eg, PET-CT) using standard phantoms, assess requirements for additional multiple and emerging modalities will be expanded. Finally, resulting data will be disseminated through a centralized web-based archive for medical and dosimetry applications for research, teaching and comparisons facilitated by collaboration with a variety of stakeholders.

Additional efforts to address other aspects of medical imaging are planned. In the field of X-ray-based bone-density measurements (DXA), the development and use of absolute, well-characterized phantoms, traceable to national standards, for confidence in calibration for a range of instruments/systems will lead to a reduction in variability among instruments due to system-specific technical and engineering issues. The development of standards for quantitative PET-MRI (to allow quantification of medical images in terms of spatial dimension and contrast/positron emission intensity) will involve the development of appropriate standard phantoms and their adoption in practice to calibrate PET-MRI scanners traceable to national standards. New standards for patient-based quantitative nuclear medicine imaging will lead to safer and more effective treatment for the over 1.4 million US patients diagnosed with cancer each year through the development of well-calibrated radioactive source standards used to obtain accurate radioactivity distributions, providing a key benchmark for analysis of patient image data, and standardized image databases for comparing internal dose models. The development of calibration standards, implementation of proficiency testing schemes, direct collaboration with clinicians, drug and instrument manufacturers to assess needs and adoption of standards in practice will address intrinsic result variability due to difference in scanner performance and overcome the limitations in the ability to discern only large changes in size/metabolism by providing standards to monitor equipment performance and support methods for “change analysis” to determine treatment efficacy.

Computed tomography and PET-CT are increasingly used to diagnose disease, to plan and monitor response to treatment and in the evaluation of drug efficacy; the usefulness of these techniques depends on consistent patient data over weeks or months, and from one clinical center to another. National measurement standards have been lacking for instrument calibrations, data acquisition protocols and data handling. In order to address this lack, this laboratory has been developing standard artefacts, including phantoms, for quantitative CT and PET, which will allow traceability of instrument calibrations back to national standards and provide a foundation for reproducible results. The development of a length standard fiducial reference phantom for CT applications, the first national calibration of ^68^Ge for PET instrument calibrations and the development of the initial anthropomorphic lung phantom, together with on-going algorithm validations (from lung cancer data) for image analysis, will impact a growing percentage of the general population. The ability to calibrate diagnostic imaging tools in a way that is traceable to national standards will lead to a more quantitative approach; both physician and patient benefit from increased accuracy in treatment planning, as well as increased safety for the patient and financial savings for the entire industry.

## Footnotes

* Certain commercial equipment, instruments, or materials are identified in this paper to foster understanding. Such identification does not imply recommendation or endorsement, nor does it imply that the materials or equipment identified are necessarily the best available for the purpose.
